# Bone marrow mesenchymal stem cell co-adjuvant therapy with albendazole for managing *Toxocara vitulorum*-rat model

**DOI:** 10.14202/vetworld.2021.347-363

**Published:** 2021-02-08

**Authors:** Faten A. M. Abo-Aziza, Abdel Kader A. Zaki, Ahmed I. Alajaji, Saleh M. Al barrak

**Affiliations:** 1 Department of Parasitology and Animal Diseases, Veterinary Research Division, National Research Centre, Cairo, Egypt; 2 Department of Physiology, Faculty of Veterinary Medicine, Cairo University, Giza, Egypt; 3 Department of Veterinary Medicine, College of Agriculture and Veterinary Medicine, Qassim University, Buraydah, Saudi Arabia

**Keywords:** albendazole, bone marrow mesenchymal stem cells, cell therapy, rat, *Toxocara vitulorum*

## Abstract

**Background and Aim:**

*Toxocara vitulorum* is a bovine intestinal nematode. Immune pictures following infection are conflicting and stopping anthelmintic albendazole treatment recording reversed liver abnormalities. The purpose of this work was to evaluate the therapeutic potential of bone marrow mesenchymal stem cells (BMMSCs) therapy, subsequent to albendazole administration in rats infected with *T. vitulorum*.

**Materials and Methods:**

The ultrasonographic and histopathological examinations as well as serum liver enzymes activity and the kinetics of recovery were investigated. The correlation of cell-mediated and humoral immune pictures was assessed by assaying immunoglobulins, splenocytes viability, phagocytic index, and Th1/Th2 cytokines.

**Results:**

The cultured BMMSCs counting were 4.21×10^4^ cells/cm^2^ with 96.03% viability. Flow-cytometric analysis indicated positive CD90 (82%), CD105 (79%) and negative CD34 (0.37%), CD45 (0.42%), attesting to the suitability of the isolated BMMSCs for use in therapy. Transplantation of BMMSCs after albendazole administration significantly reduced the release of liver enzymes (p<0.05) indicating liver cellularity improvement. The ultrasonographic, macroscopic, and histopathological findings confirmed the biochemical results. Significant elevation in the levels of tumor necrosis factor (TNF)-α and interferon (INF)-γ with a decline in interleukin (IL)-4 was observed in the untreated model (p<0.05). However, albendazole treatment followed by BMMSCs therapy significantly lowered the release of TNF-α and INF-γ, associated with significant production of IL-4 and IL-10 (p<0.05).

**Conclusion:**

The final results indicated that the liver functions, histopathological findings, and immune parameters were aggravated after experimental *T. vitulorum* infection. Albendazole treatment followed by BMMSCs therapy was found to assist in regeneration of injured hepatic tissue. Besides, it appeared to modulate host defensive immune responses against *T. vitulorum* antigens. This work could define more clearly the events that manipulate the host immune, histopathological, and biochemical responses to minimize obstacles in using stem cell therapy in animal toxocariosis.

## Introduction

Parasitic infections of bovine species are problematic worldwide diseases and are considered as the main obstacle in milk and meat production. Toxocariosis is a serious zoonotic parasitic disease with high prevalence in the tropical and subtropical regions of the world causing high morbidity and mortality rates [1]. *Toxocara vitulorum* is a common ascarid nematode affecting young cattle [2] and buffaloes [3,4]. This parasite can also infect other mammals and rodents as paratenic hosts [5]. The prevalence of *T. vitulorum* is relatively correlated to hot and humid climate [6]. The prevalence is affected by species, sex, and age of animals. In Egypt, the prevalence of *T. vitulorum* among large ruminants is considered high. One survey in the lower Egypt revealed that 47.9% of slaughtered cattle and buffalo harbored adult worms [7]. The parasite causes a variety of symptoms from anorexia, morbidity, weight loss, and even death of infected animals [8]. It also indirectly causes reduced production of milk, meat, and poor quality of skin [9] The adult female worms are 15-30 cm in length, lay eggs (8000-100,000) with a thick protective shell that provides opposition against adverse environmental conditions enabling eggs to stay alive for many years [8,10].

There is no evidence that *T. vitulorum* can induce larva migrans syndrome in humans, although it has been demonstrated that *T. vitulorum* can result in tissue migration in experimentally infected animals [9]. Studies in mice as a paratenic host showed that *Toxocara* spp. larvae penetrate the wall of the small intestine when ingested and invade multiple tissues of the body through the systemic circulation. Tissue migration has been reported to cause coagulative necrosis [11] and degenerative changes in different organs of mice [12]. According to the authors, the most commonly affected organs are the liver, lung, and eyes. Most larvae migrate directly to the liver through the portal vein; however, a few enter the mesenteric lymph nodes [13]. The migration also induces also an aggregation of inflammatory cells, mainly eosinophils which are later on replaced by macrophages and finally fibroblasts. The parasitic infection stimulates both humoral and cell-mediated immune defense [12]. However, the role of the immune response in adult worm expulsion is still unclear and the most effective responses depend on the particular parasite and the stage of infection [14]. In some conditions, the control of *T. vitulorum* is not easy as the larvae migrate in the tissues, remaining as hidden or hypobiotic worms [15].

The treatment of tissue migration continues to depend on albendazole, levamisole, and fenbendazole [2]. Albendazole is a benzimidazole carbamate, which is considered as the drug of choice for animal toxocariosis [16]. Yet, one of the drawbacks of albendazole is its low water solubility, which may be the major problem retarding its systemic use [17]. It was reported that stopping of albendazole treatment resulted in reversing liver abnormalities [17]. This motivates the development of new strategies to increase its therapeutic efficiency. Stem cell therapy is a sort of interference strategy that inserts new cells into deteriorated tissues, that assist the treating of various diseases and injuries [18,19]. In addition, stem cell therapy has been successfully used in the treatment of parasitic infections in experimental animals and acceptable results have been achieved [20]. Previous successes of cell therapy in infectious diseases and its anti-fibrotic properties were reported [21,22]. In this context, this study aimed to evaluate the potential of the complementary combination of albendazole as anthelmintic drug and (expand) bone marrow mesenchymal stem cells (BMMSCs) therapy for treating *T. vitulorum* rat model. Liver state of treated rats was assessed by ultrasonographic and histopathological techniques as well as serum protein profile and liver enzymes activity. Cell-mediated response and humoral immunomodulation were examined by analyzing the levels of immunoglobulins and leukocytes, splenocytes viability, phagocytic index, and Th1/Th2 cytokines quantification.

## Materials and Methods

### Ethical approval

This study was strictly performed according to the recommendations in the Guide for the Care and Use of Laboratory Animals of the National Institutes of Health. The protocol was approved by the Institutional Animal Care and Use Committee of National Research Centre, Cairo, Egypt (Protocol No: 19/151). All procedures, including animals were carried out under anesthesia and all efforts have been made to reduce suffering.

### Study period and location

The study was conducted from July 2019 to March 2020 at the Central Laboratory of Veterinary Research, National Research Centre, Cairo, Egypt.

### Isolation and characterization of rat BMMSCs

#### Isolation of rat BMMSCs

The BMMSCs were isolated and expanded from cells isolated from the femur bone marrow of healthy albino rats using Ficoll density gradient 1.077 (Ficoll-HyPaque) [23]. The mononuclear cells obtained were suspended in alpha minimum essential medium (α-MEM, Sigma-Aldrich, USA) complemented with 20% fetal bovine serum (FBS, Sigma-Aldrich, USA), 2 mM L-glutamine (Sigma-Aldrich, USA), 55 μM 2-mercaptoethanol (Sigma-Aldrich, USA), and two antibiotics; penicillin (100 U/mL) and streptomycin (100 μg/mL). The suspended cells were cultured into 100 mm polystyrene plastic tissue-culture dishes at a density of 10×10^6^ (Greiner Bio-One, Germany) and maintained at 37°C and 5% CO_2_ incubation. Non-adherent cells and hematopoietic stem cells were discarded and the medium was changed every 3 days. On reaching 80% confluence, the cells were dissociated and collected by the addition of 0.25% (w/v) trypsin-EDTA solution (Sigma-Aldrich, USA) and seeded in 25 cm^2^ plastic cell culture flasks (Greiner Bio-One, Germany) at a density of 10^4^ cells and serial passaging was done. The cells harvested at passage four (P4) were subjected to cell characterization and proliferation assays.

### Cell counting and viability of BMMSCs

Characterization of BMMSCs was done at the P4. The harvested cells were centrifuged at low speed for 5 min. The cells were then resuspended in 2 ml α-MEM and were counted. BMMSCs viability was assessed in percentage using trypan blue stain exclusion method [24].

### Phenotyping of BMMSCs

Flow cytometry for BMMSCs surface antigen phenotyping was performed as described previously [25]. Briefly, 2×10^5^ cells were used and incubated with phycoerythrin (PE) conjugated antibodies specific for rat CD34, CD45, CD90, and CD105 (BD Biosciences, US). Stained cells were measured by a FACS Calibur flow cytometer (BD Biosciences, US). Negative sample untreated control isotype was included in all analyses.

### Colony-forming units (CFUs) assay of BMMSCs

CFUs assay was performed to determine BMMSCs proliferation capacity. A total of 10^4^ cells from the P4 were plated in culture dish (100 mm). Washing was done on reaching 80% confluence and the cells were observed and counted using an inverted microscope. Each group of cells containing over 50 cells was counted as a colony [26].

### Bromodeoxyuridine (BrdU) assay of BMMSCs

BMMSCs proliferation was assessed by BrdU incorporation assay as previously described [26]. Each cell population was seeded on two well chamber slides (Nunc) at a density of 1×10^4^ cells/well and cultured for 2-3 days. Then, after 20 h incubation with 1:100 BrdU solution cultured cells were stained using a BrdU staining kit (Invitrogen). To determine BMMSCs proliferation capacity, BrdU positive and total cell numbers were counted in ten images. The proliferation was exposed as BrdU positive cells percentage over the total cells.

### Population doubling (PD) assay of BMMSCs

PD score and PD time (PDT) assays were calculated [27]. A total of 0.25×10^6^ cells were seeded on 75 cm^2^ tissue culture flasks. Cells were harvested at 80% confluence using Trypsin-EDTA to be passaged until the cell division cease. The cells were observed and counted at each passage using inverted microscope and each PD was determined using the equation of log^2^ (number of harvested cells at the end of incubation/number of seeded cells at the beginning of incubation). The final PD score was calculated by accumulative adding of total numbers of every passage until the stopping of cell division. PDT was recorded in P1-P2, P5-P6, and P11-P12 by the following equation: PDT=1/[3.23 (log Harvested cell number–log number of inoculated cell)/(t_2_–t_1_)]. Where, t_1_: time at inoculation, t_2_ time between inoculation and harvesting.

### Preparation of *T. vitulorum* antigens

Male and female adult *T. vitulorum* were collected from the small intestine of naturally infested buffalo-calves immediately after slaughter. Five antigens of *T. vitulorum* were prepared as per standard protocol [28]. For *T. vitulorum* excretory-secretory (Tv-ES) antigens, the collected *T. vitulorum* worms were rinsed several times with phosphate buffer saline (PBS), pH 7.2 containing 1:1 penicillin and streptomycin and incubated overnight in RPMI at 27°C. The medium was collected and saturated with solid ammonium sulfate then centrifuged at 4000 rpm for 15 min. The precipitate was dissolved in PBS and dialyzed against distilled water, then centrifuged at 14,000 rpm in a cooling centrifuge at 4°C for 20 min. The supernatant was stored at −20°C as Tv-ES antigen. The *T. vitulorum* crude somatic (Tv-CS) antigen was prepared by rinsing adult worms several times before homogenization in PBS. Three cycles of sonication was done (0.5 amplitude, 1 min) followed by centrifugation at 14,000 rpm in a cooling centrifuge at 4°C for 30 min. The supernatant was aspirated and kept at −20°C as Tv-CS antigen. The uteri, digestive tubes, and cuticles were obtained separately after dissection of ten adult worms. To prepare *T. vitulorum* egg (Tv-E) antigen, eggs were released by gentle pressure on the separated uteri of the adult female worms and resuspended in PBS. Eggs were washed several times with 0.01 PBS by centrifugation at 1500 rpm for 3 min. The sediment eggs were mixed with an equal volume of the same solution then sonicated for three cycles (0.5 amplitude, 1 min) until no intact eggs were microscopically visible and became homogenous. The homogenate was centrifuged in cooling centrifuge at 4°C for 30 min at 14,000 rpm. The supernatant was aspirated and kept at −20°C as Tv-E antigen. The digestive tubes were collected and homogenized in PBS and cooling centrifugation was done at 4°C for at 30 min at 14,000 rpm. The supernatants were aspirated and kept at −20°C as *T. vitulorum* digestive tract content (Tv-DTC) antigen. The collected cuticles of ten worms were macerated and homogenized in PBS followed by three cycles of sonication (0.5 amplitude, 1 min) and centrifugation in a centrifuge at 14,000 rpm for 30 min. The supernatant was aspirated and kept at −20°C as *T. vitulorum* cuticle (Tv-C) antigen. Total protein contents of the different antigens were determined colorimetrically using SPECTRUM kits (BioMerieux, SA).

### Preparation and potency assay of immunoglobulins against isolated *T. vitulorum* antigens

Fifteen healthy white New Zealand male rabbits with body weight 2.0 kg each, were used (n=3/antigen) to prepare immunoglobulins against *T. vitulorum* prepared antigens as described previously [29]. Briefly, blood samples were collected from each rabbit before experimentation. Sera were separated and kept at –20°C as control negative. Each antigen was calibrated to contain 1 mg/mL. Emulsion of 2 mL of each calibrated antigen was done using an equal volume of complete Freund’s adjuvant (Sigma-Aldrich, USA). The emulsified antigen was intradermally injected weekly at several sites for 4 successive weeks. Final booster dose from each incomplete Freund’s adjuvant (Sigma-Aldrich, USA) emulsified antigen was injected subcutaneously. One week later, the immunoglobulin levels in the rabbit sera were estimated by testing using agar gel diffusion test as previously described [30]. When a distinct precipitin line was found in the gel, it was considered strongly positive results and the rabbits were slaughtered and sera were collected from each and kept at –20°C.

The potencies of the prepared immunoglobulins against the prepared antigens were evaluated using solid-phase enzyme-linked immunosorbent assay (ELISA) [29]. Calibration of the immunoglobulins values was performed based on the previous method using serum collected from infected and non-infected calves to be used as positive and negative references respectively [31]. The concentrations of immunoglobulins against different antigens were used. Briefly, each antigen was diluted with carbonate buffer (pH 9.6) to coat the wells, followed by overnight incubation at 4°C. Blocking of wells was done for an hour using 1% bovine serum albumin (pH 9.6), followed by addition of diluted sera (1:50, with 0.05% PBS-T-20) and incubated for 2 h. Wells were washed and protein-A-peroxidase conjugate (100 μL/well) was added and then incubated at 37°C for 1 h. Ortho phenylenediamine (OPD, 20 mg/ml) was added as a substrate solution and the optical densities (ODs) were measured using MR 700 Microplate ELISA reader (USA) at a wave length of 450 nm.

### *T. vitulorum*-rat model

**Figure-1:**
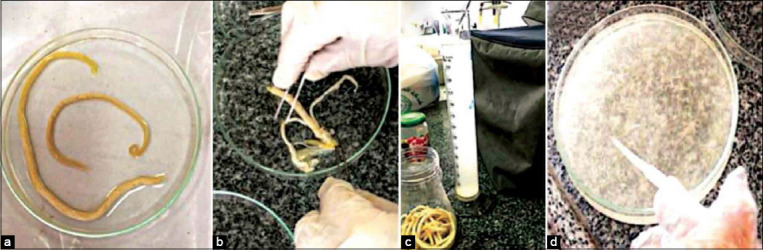
Collection of *Toxocara vitulorum* egg. (a) Collected *T. vitulorum* from the jejunum of naturally infected buffalo calves. (b) Eggs were collected from the adult female worms’ uteri and (c) leave for sedimentation in normal saline. (d) The collected eggs were incubated in Petri dishes with 0.5% formalin solution at room temperature for 15-45 days with infrequent agitation and inspection.

**Figure-2:**
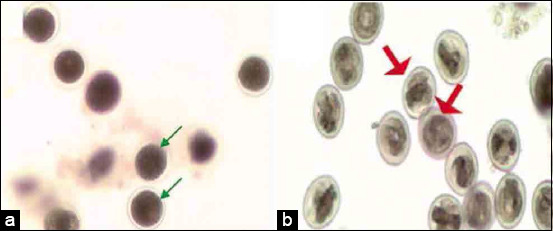
Light micrographs for non-embryonated egg and embryonated egg of fertilized *Toxocara vitulorum* egg. (a) Non-embryonated egg (green arrow) and (b) embryonated egg with larva (red arrow) 10×.

The eggs collected from *T. vitulorum worms* were incubated in Petri dishes with 0.5% formalin solution at room temperature (Figure-1) with infrequent agitation and examination for 15-45 days [32]. The embryonated egg suspension was rinsed by clean tap water several times. Preparation of the infective inocula was done by determining the number of ova in samples of 20-mL of ova stock suspension using McMaster slide until 96% of the eggs were shown to contain larvae (Figure-2). The viability of the larvae was evaluated immediately before infection by movement monitoring. Twenty-four adult male albino rats with body weight 120 ± 5.0 g were used. Six rats were considered as healthy control. The remaining 18 rats were experimentally infected with *T. vitulorum* as described previously [12] with some changes in the dose of egg. Infection of rats was induced by giving an oral dose of 800 embryonated eggs in 2 mL water and kept for 8 weeks with the consistent clinical signs examination. All animals were housed with adequate hygienic conditions and provided water and ration *ad libitum*.

### Experimental design

At the end of the 8 weeks of experimental infection, the healthy control rat group (n=6/group) was injected with Dulbecco’s Modified Eagle Medium (DMEM; Sigma-Aldrich, USA) supplemented with 2 mM L-glutamine and 20% FBS. The other three *T. vitulorum*-rat model groups (n=6/group) were considered as 2^nd^, 3^rd^, and 4^th^ groups. The 2^nd^ group was considered as untreated *T. vitulorum*-rat model and injected with DMEM. The 3^rd^ group was considered as albendazole treated group and orally administered with a dose of 15 mg/kg/day albendazole (E.I.P.I.Co. Pharmaceuticals) suspended in DMEM twice daily for 14 days [33]. The 4^th^ group was transplanted with BMMSCs following albendazole treatment (albendazole with BMMSCs). Transplantation of BMMSCs into *T. vitulorum-*rat model was done after albendazole treatment by a single 0.5 ml suspended dose of 1.5×10^6^ cells/rat through the hepatic area [20]. Briefly, the abdominal hair of each rat was removed and the skin was disinfected with 70% alcohol. The upper right quadrant at the liver area was well-defined and a divided dose was injected directly into this area over 15 min. At the 2^nd^ and 4^th^ weeks post treatment, liver injuries and blood flow were monitored by ultrasonography (see following section). Blood smears from each individual, stained by Giemsa stain after 2-3 min fixation in methyl alcohol was examined for differential count of leukocytes. Sera were extracted from blood samples by centrifugation at 3000 rpm for 15 min to be used for biochemical and immunological evaluation. Body weight, temperature, and behavioral changes were daily monitored during the experiment to be used as specific endpoint criteria to determine when animals should be euthanized. Once animals reached endpoint criteria, such as abnormal body temperature, body weight, behavioral changes, or pathological changes, they were immediately euthanized for a humane death. At the end of the experiment, the animals (n=24) were euthanized using sodium pentobarbital anesthesia and their livers and spleen were collected in 10% formalin for histopathology and preparation of splenocytes, respectively. For humane endpoints, the guidelines of National Centre for the Replacement Refinement and Reduction of Animals in Research were followed by the authors who are well trained for animal care and handling. All animal welfares measures were taken into consideration to minimize suffering and distress, using sodium pentobarbital anesthesia during the experiment.

### Ultrasonographic examination

Ultrasonographic examination was carried out at the 2^nd^ and 4^th^ weeks post-treatment to detect the rats with focal lesions [34]. After anesthetized using 50 mg/kg ketamine and 10 mg/kg xylazine, the upper right quadrant abdomen of all rats was clean-shaven. The livers were examined using pulsed-wave Doppler ultrasound scanner (SonoAce R3, Medison, Samsung, South Korea) equipped with a linear-array 12 MHz transducer to demonstrate the site, edges, maximum size, and echogenicity of lesions. Blood flow of the liver was measured for every rat by color Doppler ultrasonography by color and power flow modes. Six Hz pulse-repetition frequency and identical color gain settings were used for all scans. The spectral mode was activated after the observation of the cardiac cycle in the hepatic artery. For tracking the blood flow, the peak systolic velocity, end diastolic velocity (EDV), time average maximum velocity (TAMV), systolic/diastolic (S/D), resistance index (RI), and pulsatility index (PI) were measured.

### Histopathological examination

Collected liver tissue specimens were used for the preparation of paraffin block after fixation in 10% neutral buffered formalin. Thin slices of 4-5μm thickness were then processed and stained with hematoxylin and eosin to examine the changes microscopically [35].

### Biochemical analysis

Biochemical parameters in sera were determined at 2^nd^ and 4^th^ weeks post-treatment. Total protein and albumin were determined colorimetrically using SPECTRUM kits (BioMerieux, SA). Calculation of globulin and albumin/globulin ratio was done. Cholesterol, alanine aminotransferase (ALT), aspartate aminotransferase (AST), and alkaline phosphatase (ALP) were assayed by specific Linear Chemicals S.L. kits.

### Determination of humoral response

The sera of different groups of rats were subjected to an evaluation of immunoglobulins content using solid-phase ELISA [36]. Briefly, each prepared antigen (Tv-ES, Tv-CS, Tv-E, Tv-DTC, and Tv-C) was diluted in PBS to the desired concentration. A volume 100 μL/well of each antigen was added in the ELISA plate wells and incubated for 12 h at 4°C. Bovine serum albumin (1%) was used to block the unattached spots at 37°C for 1 h. Serial dilution of 100 μL/well of serum samples was added after blocking and incubated at 37°C for 2 h then at 4°C for 12 h. Checker-board titration (1:2000) of enzyme conjugate horseradish peroxidase-labeled goat anti-mouse immunoglobulin (Sigma-Aldrich, USA) was done and add to the plates for 1 h at 37°C followed by 100 μL peroxidase substrate to each well. The plates were then covered and kept at room temperature for 45 min. OPD solution was added and incubated in the dark at room temperature for 7 min. The reaction was stopped using 50 μL/well of 2.5M H_2_SO4 (Sigma-Aldrich, USA). The intensity of color obtained was measured at 450 nm using a microplate ELISA reader (Dynatic product, USA). Total immunoglobulins anti-Tv-ES, Tv-CS, Tv-E, Tv-DTC, and Tv-C antibodies were expressed as ODs.

### Determination of cell-mediated immune response

#### Leukogram

The % of eosinophil, neutrophil, and lymphocyte were counted by cross edges method and neutrophil/lymphocyte (N/L) ratio was calculated [37].

### Spleen lymphocytes viability

Lymphocytes of spleen were prepared as described previously [38]. The collected spleen specimens were suspended in ice-cold α-MEM supplemented with 5% FBS. Splenocytes were then released by meshing the specimens with tweezers and washed 2 times in α-MEM. A total of 200 μL blood/2 mL warm isotonic ammonium chloride lysis buffer solution (0.155 M NaH_4_Cl, 0.01 M KHCO_3_, and 0.1 mM EDTA diluted with distilled water 1:10 before use) was used for lysing erythrocytes. After centrifugation, the cells were resuspended in α-MEM medium. The viability of splenocytes was determined using trypan blue dye exclusion method [24].

### Phagocytic index

The phagocytic index of T cells in the blood was evaluated using *in vitro* carbon clearance assay [37]. Briefly, 5 μL Indian ink was added to 1.5 mL from each blood sample. Each mixture was divided into three aliquots which were diluted with 3 mL saline and incubated at 37°C for 2 times (20 and 40 min). The incubated samples were centrifuged at low speed for 5 min and the supernatant was collected. The OD was read using a spectrophotometer at 535 nm, with the distilled water taken as blank. The phagocytic index was calculated by converting the OD (log^2^) on time (h).

### Th1/Th2 cytokines

Assay of Th1 cytokines (tumor necrosis factor [TNF]-α and interferon [INF]-γ) and Th2 cytokines (interleukin [IL]-4 and IL-10) levels in the sera sample at 2^nd^ and 4^th^ weeks post-treatment was performed using ELISA kits (Sigma-Aldrich, USA). The manufacturer’s instructions were followed and the color change was measured at 450 nm using microplate ELISA reader (Dynatic product, USA).

### Statistical analysis

The values of results were tabulated as means ± standard errors. Descriptive statistical analysis of data was performed using one-way ANOVA test by SPSS version 20.0 (IBM Corp. NY, USA). The degree of the probability (p) <0.05 and 0.01 was considered as significantly different. For the liver ultrasonography, Duncan’s multiple range test was used.

## Results

### Counting and viability of BMMSCs

**Figure-3:**
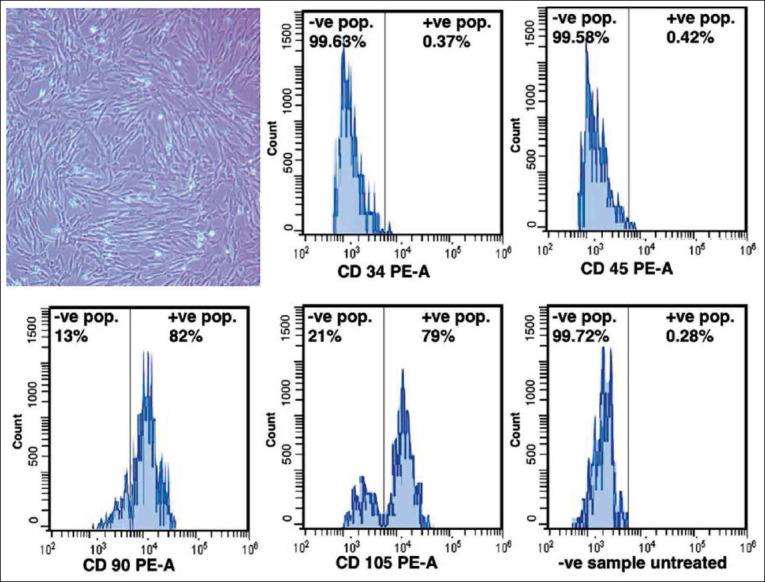
Isolation and phenotypic analysis of bone marrow mesenchymal stem cells (BMMSCs). (a) Photomicrograph of cultured BMMSCs reached 80% confluence showing spindle and stellate shaped appearance 10×. Phenotypic analysis showed that cultured BMMSCs positively expressed CD90 (82%) and CD105 (79%) and negatively expressed CD34 and CD45 antibodies staining. Negative sample untreated showed 0.28 +ve population and 98.52 –ve population.

BMMSCs of rats were isolated from bone marrow and cultured to adhere on the bottom of the culture dishes. After 1-2 days from initial seeding, cells were observed by an inverted microscope and it began to form process and some cells became spindle-shaped and appeared as fibroblasts. BMMSCs continued to grow and multiply up to 80% confluence after 14 days from initial seeding (Figure-3a). On reaching 80% confluence, the cell count was 4.21×10^4^ cells/cm^2^ with 96.03±3.72% viability (Table-1).

**Table-1:** Cell counting, viability, final PD and CFUs of rat BMMSCs.

Cell count (cells/cm^2^)	Viability (%)	Final PD score	CFUs
4.21×10^4^	96.03±3.72	76.82±5.95	22.11±1.17

CFU=Colony-forming units, PD=Population doubling, BMMSCs=Bone marrow mesenchymal stem cells

### Phenotypic analysis

By flow cytometric analysis, BMMSCs expressed MSC markers (Figure-3). Particularly, BMMSCs positively expressed CD90 (82%) and CD105 (79%) and negatively expressed CD34 (0.37%) and CD45 (0.42%).

### Proliferation of BMMSCs

**Figure-4:**
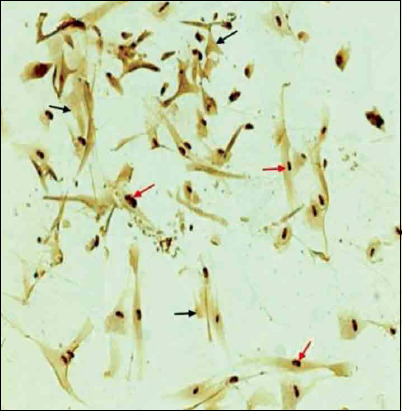
Cell proliferation assays. BrdU incorporation of BMMSCs for 24 h was evaluated. BrdU positive cells with brown stained nuclei (red arrows) and negative cells were unstained (black arrows). BrdU=Bromodeoxyuridine, BMMSCs=Bone marrow mesenchymal stem cells.

**Figure-5:**
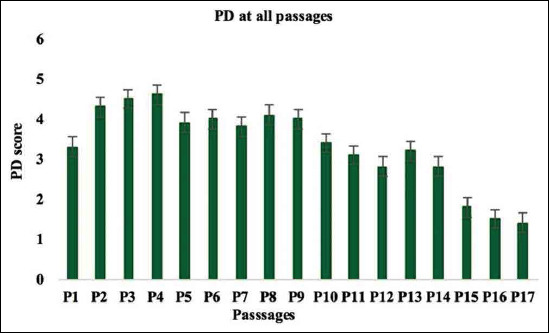
Cell proliferation evaluation of BMMSCs by PD assay. PD was significantly increased on reach P9 then declined until the cells division stopped at P17. Error bars represent Mean ± SE. PD=Population doubling, BMMSCs=Bone marrow mesenchymal stem cells.

**Figure-6:**
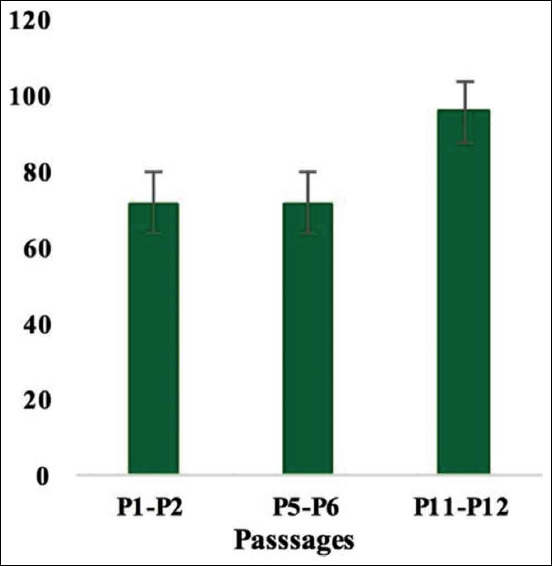
PDT of BMMSCs in P1-P2, P5-P6, and P11-P12 were recorded. PDT significantly higher in P11-P12 than that in other passages. Error bars represent Mean ± SE. PDT=Population doubling time, BMMSCs=Bone marrow mesenchymal stem cells.

The percentage of BrdU positive cells was significantly increased on reaching 80% confluence (Figure-4). The results of the PD revealed that BMMSCs showed maximal expansion potential in P3 and P4 and exhibited a significant increase in total PD. This elevation continued until P9; however, it started to decline at P10 until the cell growth was arrested in P16 and P17 (Figure-5). Each PDT was analyzed in P1-P2, P5-P6, and P11-P12, respectively. In these passages, the PDT of P1-P2 and P5 to P6 was shorter than P11-P12 (Figure-6). Moreover, BMMSCs at 80% confluence showed 76.82±5.95 final PD score (Table-1). *In vitro* study of CFUs count revealed that the quantity 22.11±1.17 (Table-1).

### Immunoglobulins response of *T. vitulorum*-rat model and treatment

**Figure-7:**
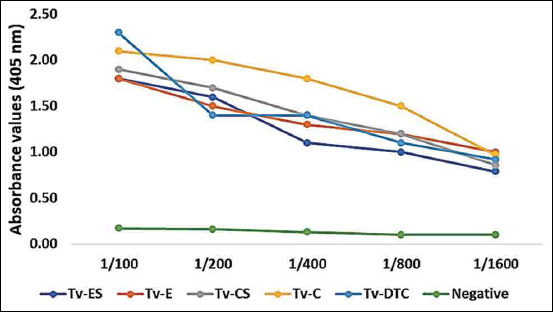
Screening of enzyme-linked immunosorbent assay calibration. Negative calves’ sera (n=5) and positive calves’ sera (n=9) were calibrated against five protein antigens of *Toxocara vitulorum* at a concentration of 10 μg/mL.

The slope of regression (b) among the graded log-doses of Tv-ES, Tv-CS, Tv-E, Tv-DTC, and Tv-C antigens ODs was 0.152, 0.312, 0.265, 0.174, and 0.131, respectively. ELISA calibration of negative and positive calves’ sera against the five protein antigens at a concentration of 10 μg/ml was justified (Figure-7). Anti-*Toxocara* immunoglobulins antibody titers in serum of the healthy control rats, untreated and treated *T. vitulorum*-rat models were determined using solid-phase ELISA technique against five *Toxocara* antigens (Table-2). A significant increase (p<0.01) in immunoglobulins antibody titers against all *T. vitulorum* antigens was observed in the *T. vitulorum*-rat model relative to the healthy control by 12 weeks after infection. In the group treated with albendazole, there was a significant drop (p<0.05) in antibody level against Tv-CS, Tv-C, and Tv-DTC parasite antigens at the 4^th^ week post-treatment. However, in the rats transplanted with BMMSCs in combination with albendazole, there was a significant drop (p<0.05) in antibody titer against two parasite antigens (Tv-E, Tv-C, and Tv-DTC) at the 2^nd^ week post-treatment. This drop became more pronounced and significant (p<0.01) at the 4^th^ week post-treatment against all prepared *Toxocara* antigens in rats transplanted with BMMSCs in combination with albendazole.

**Table-2:** Immunoglobulins against Tv-ES, Tv-E, Tv-CS, Tv-C, and Tv-DTC antigens in the serum of healthy control, untreated and treated *T. vitulorum*-rat models (OD).

Antigens	Healthy control	*T. vitulorum-rat models*				

		Untreated	Albendazole		Albendazole with BMMSCs	
	
			2^nd^ week	4^th^ week	2^nd^ week	4^th^ week
Tv-ES	0.043±0.01	0.429^a^±0.06	0.416±0.05	0.335±0.06	0.371±0.05	0.226*±0.05
Tv-E	0.102±0.03	0.507^a^±0.05	0.491±0.05	0.372±0.07	0.138*±0.04	0.309*±0.04
Tv-CS	0.049±0.01	0.648^a^±0.04	0.532±0.11	0.422*±0.07	0.475±0.11	0.323*±0.12
Tv-C	0.113±0.03	0.778^a^±0.04	0.636±0.12	0.426*±0.13	0.511*±0.09	0.371**±0.09
Tv-DTC	0.133±0.03	0.781^a^±0.02	0.672±0.14	0.438*±0.11	0.512*±0.05	0.264**±0.05

Mean±SE. Values having letter

a are significantly different than corresponding healthy control at p<0.01. Values having

*, ** are significantly different than corresponding untreated at p<0.05 and p<0.01, respectively.

*T. vitulorum*=*Toxocara vitulorum*, Tv-ES=*T. vitulorum* excretory-secretory, Tv-E=*T. vitulorum* excretory-secretory, Tv-CS=*T. vitulorum* crude somatic, Tv-C=*T. vitulorum* crude somatic, Tv-DTC=*T. vitulorum* digestive tract content, OD=Optical densities, BMMSCs=Bone marrow mesenchymal stem cells

### Ultrasonography

**Figure-8:**
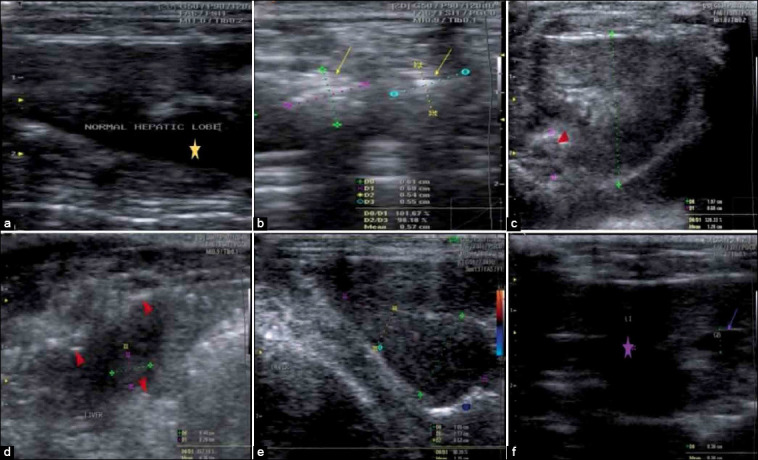
Ultrasonographic hepatic pictures in different *Toxocara vitulorum*-rat models. (a) Normal liver ultrasonography image was observed in healthy control rats showing homogeneous parenchyma, with medium echogenicity level and a regular surface of liver (yellow star). (b) Liver of untreated *T. vitulorum*-rat model with diffusely higher echogenicity of heterogeneous parenchyma and multiple foci of fibrosis (yellow arrows) with average size 0.57 cm. (c) At the 2^nd^ week following albendazole treatment, a hypoechoic regenerative nodule (red arrowhead) with a mean diameter reached 0.6 cm. (d) At the 4^th^ week following albendazole treatment, the liver ultrasonography image showed hyperechoic of the parenchyma and liver surface appeared as a slightly irregular line (red arrowheads). (e) At the 2^nd^ week after albendazole treatment with BMMSCs transplantation, ultrasonography image showed normal liver lobe of rats with homogeneous parenchyma, medium level echogenicity and regular surface, diameter 1.05×2.17×0.52. (f) At the 4^th^ week after albendazole with BMMSCs transplantation, liver showed an improvement in the hepatic lobe presented by homogeneous hepatic parenchyma with medium level echogenicity and regular surface (violet star) and gall bladder (violet arrow). BMMSCs=Bone marrow mesenchymal stem cells.

In Figure-8, ultrasonographic images revealed liver of untreated *T. vitulorum*-rat model with diffusely hyperechoic of heterogeneous parenchyma with multiple foci of fibrosis with average size of 0.57 cm. In the 2^nd^ week following albendazole treatment, a hypoechoic regenerative nodule with mean diameter reached 0.6 cm was observed. However, in the 4^th^ week following albendazole treatment, the hyperechoic regions of the parenchyma and liver surface appeared as a slightly irregular line. Moreover, in the 2^nd^ week after albendazole treatment with BMMSCs transplantation, the ultrasonography image demonstrated normal liver lobe of rats with homogeneous parenchyma showing medium echogenicity level and regular surface. In the 4^th^ week after albendazole with BMMSCs transplantation, liver showed an improvement and the hepatic lobe showed a homogeneous hepatic parenchyma with medium level echogenicity and regular surface and gall bladder.

**Figure-9:**
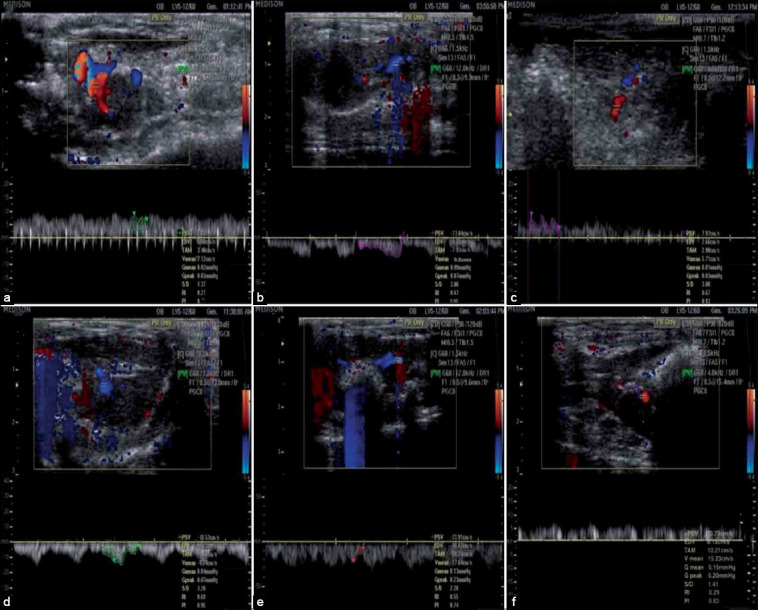
Color and spectral Doppler ultrasonography portal vein waveforms. (a) Portal vein of healthy control rats showing continuous hepatopetal flow toward the liver demonstrated in red on the color Doppler sonogram and antegrade flow above the baseline in the spectral waveform with normal V mean (17.12 cm/s) and moderate PI (0.71). (b) Portal vein of *Toxocara vitulorum*-rat model showing slow, hepatofugal flow away from the liver as blue in the color Doppler sonogram and as retrograde flow below the baseline in the spectral waveform with low V mean (4.3 cm/s) and PI (1.74). (c) Portal vein at the 2^nd^ week following albendazole treatment showing hepatopetal flow toward the liver as red in the color Doppler sonogram and antegrade above the baseline in the spectral waveform with low V mean (5.71 cm/s) and PI (0.93). (d) Portal vein at the 4^th^ week following albendazole treatment showing hepatofugal flow away from the liver as blue in the color Doppler sonogram and retrograde flow below the baseline in the spectral waveform with low V mean (9.74 cm/s) and PI (0.95). (e) Portal vein at 2^nd^ week after albendazole treatment with BMMSCs transplantation showing slow hepatofugal flow away from the liver as blue in the color Doppler sonogram and retrograde flow below the baseline in the spectral waveform with normal V mean (17.46 cm/s) and mild PI (0.74). (f) Portal vein at 4^th^ week after albendazole treatment with BMMSCs transplantation showing hepatopetal flow toward the liver as red in the color Doppler sonogram and antegrade flow above the baseline in the spectral waveform with mildly V mean (15.23 cm/s) and moderate PI (0.83). V=Velocity, PI=Pulsatility index, BMMSCs=Bone marrow mesenchymal stem cells.

Color and spectral Doppler ultrasonography were used to examine hepatic blood flow in different groups (Figure-9). Colored areas represent the flow velocity exceeding 10 mm/s. Normal portal vein waveform of healthy control rats was observed with continuous antegrade and hepatopetal flow as well as normal mean velocity (17.12 cm/s) and moderate PI (0.71). However, slow retrograde, hepatofugal waveform, and low mean velocity (4.3 cm/s) below the lower limit of normal with PI (PI=1.74) of the portal vein was observed in untreated *T. vitulorum-*rat model. In the 2^nd^ week following albendazole treatment, antegrade, and hepatopetal flow with low mean velocity (5.71 cm/s) and PI (PI=0.93) was noticed in portal vein waveform of infected rats. At the 4^th^ week following albendazole treatment, the portal vein waveform was observed with retrograde and hepatofugal flow and low mean velocity (9.74 cm/s) and PI (PI=0.95). However, at 2^nd^ week after albendazole treatment with BMMSCs transplantation, portal vein waveform of rats showed slow retrograde and hepatofugal flow with a mean velocity (17.46 cm/s) below the lower limit of normal and mild PI (PI=0.74). Moreover, portal vein waveform of rats at 4^th^ week after albendazole treatment with BMMSCs transplantation showed antegrade and hepatopetal flow with mildly mean velocity (15.23 cm/s) and moderate PI (PI=0.83).

### Hepatic blood flow ultrasonography analysis of untreated and treated *T. vitulorum* rat models

The ultrasonography analysis showed a picture of low hepatic blood flow in untreated *T. vitulorum-*rat model as indicated by a significant low PSV, TAMV, V (p<0.0001) and significant high EDV (p<0.0001), S/D, PI (p<0.001), RI, G peak (p<0.05), and G mean (p<0.01) compared to healthy control rats. At the 2^nd^ and 4^th^ weeks of albendazole with BMMSCs therapy, PSV elevated while EDV declined (p<0.0001) in comparison to untreated *T. vitulorum-*rat model. TAMV and V were significantly elevated in the 2^nd^ and 4^th^ weeks of albendazole with BMMSCs therapy than untreated *T. vitulorum-*rat model (p<0.0001). S/D was significantly increased in the untreated *T. vitulorum-*rat model and decreased in the 2^nd^ and 4^th^ weeks of albendazole with BMMSCs therapy. A significant decline was noticed in RI in the 4^th^ week after albendazole with BMMSCs therapy. However, a significant decline in PI was noticed in rats at both the 2^nd^ and 4^th^ weeks of albendazole with BMMSCs therapy comparing to untreated *T. vitulorum-*rat model. G mean and G peak were increased in the untreated *T. vitulorum-*rat model and declined in rats at the 2^nd^ and 4^th^ weeks of albendazole with BMMSCs therapy (Table-3).

**Table-3:** Hepatic blood flow ultrasonographic parameters of *T. vitulorum* experimentally infected and treated rats.

Parameters	Healthy control	*T. vitulorum*-rat model					p-value

		Untreated	Albendazole		Albendazole with BMMSCs		
	
			2^nd^week	4^th^week	2^nd^week	4^th^week	
PSV (cm/s)	44.15^a^±5.25	12.31^b^±3.72	17.53^b^±1.28	19.22^b^±2.45	32.78^a^±1.84	43.95^a^±1.62	0.0001
EDV (cm/s)	5.43^a^±0.17	9.29^b^±0.71	8.77^bc^±1.20	8.36^bc^±0.64	6.12^ac^ ±0.23	5.18^ac^±0.29	0.0001
TAMV (cm/s)	8.65^a^±0.32	3.77^b^±0.19	4.25^b^±0.51	3.83^b^±0.32	6.59^a^±0.42	7.43^a^±0.28	0.0001
V (cm/s)	18.46^a^±0.57	9.73^b^±0.83	10.33^b^±1.14	10.41^b^±1.27	17.55^a^±0.82	17.93^a^±0.67	0.0001
S/D	0.09^a^±0.03	0.27^b^±0.02	0.29^b^±0.04	0.25^bc^±0.06	0.14^ac^±0.04	0.11^a^±0.03	0.001
RI	0.63^a^±0.11	1.12^b^±0.14	0.97^a^±0.31	1.32^b^±0.43	0.94^a^±0.33	0.69^a^±0.09	0.05
PI	0.74^a^±0.17	1.51^b^±0.35	1.33^bc^±0.26	1.98^b^±0.16	0.85^ac^±0.18	0.72^a^±0.16	0.001
G Mean (mmHg)	0.31^a^±0.09	0.96^b^±0.11	0.92^bc^±0.13	0.72^bc^±0.11	0.49^ac^±0.09	0.37^ac^±0.11	0.01
G Peak (mmHg)	4.82^a^±0.33	8.45^b^±1.12	8.54^bc^±0.71	9.38^bc^±1.52	5.37^ac^±0.16	5.39^ac^±0.37	0.05

Mean ± SE. In the same row, values with different superscripts are significantly different. BMMSCs=Bone marrow mesenchymal stem cells, PSV=Peak systolic velocity, EDV=End diastolic velocity, TAMV=Time average maximum velocity, V=Velocity mean, S/D=Systolic/diastolic, RI=Resistance index, PI=Pulsatility index, *T. vitulorum=Toxocara vitulorum*

### Macroscopical examination of liver

**Figure-10:**
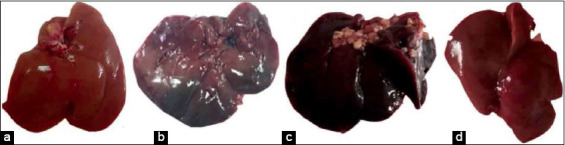
Macroscopical examination of liver in different *Toxocara vitulorum*-rat models. (a) Normal appearance of the liver of healthy control rat. (b) Hepatomegaly with sever congested dark borders were noticed in the liver of the untreated *T. vitulorum*-rat model. (c) Enlarged and congested liver of rats at 4^th^ week following albendazole administration. (d) Normal size and appearance of rat livers at the 4^th^ week after albendazole with BMMSCs therapy. BMMSCs=Bone marrow mesenchymal stem cells.

The healthy control rats showed a normal appearance of liver surfaces. However, hepatomegaly with sever congested dark borders was noticed in the livers of the untreated *T. vitulorum*-rat model. Reduction of hepatomegaly and congestion was noticed in rats at the 4^th^ week following albendazole administration. Normal appearance of the livers with approximately normal size was observed in the rats at the 4^th^ week after albendazole with BMMSCs therapy (Figure-10).

### Histopathological examination of liver

**Figure-11:**
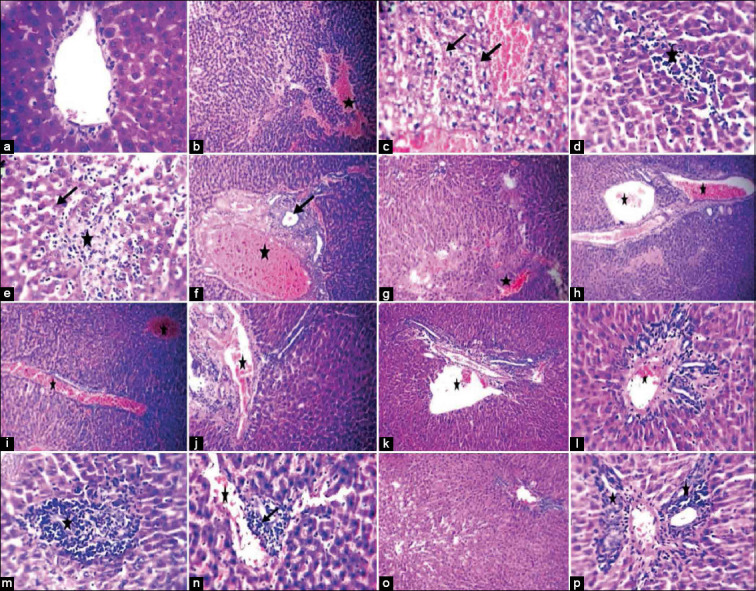
Histopathological examination of liver in different *Toxocara vitulorum*-rat models. Liver slides of healthy rats showing: (a, 20×) Apparently normal hepatocytes and central vein. (b, 10×) Liver sections of untreated *T. vitulorum*-rat model showing: Congestion of sinusoids and a wide area of hemorrhage (asterisk). (c, 20×) congestion and dilatation of sinusoids with micro and macrovesicular steatosis (arrows). (d and e, ×20) a focal area of hepatic necrosis (arrow) with leukocytic infiltration (asterisk). (f, 10×) congestion of sinusoids and portal veins (asterisk) and biliary hyperplasia (arrow). (g, 10×) Liver sections of rats at 4^th^ week following albendazole therapy showing: Congestion of sinusoids and hemorrhage (asterisk) with hepatic necrosis. (h, 10×) congestion of portal veins (asterisk). (i, 10×) congestion of sinusoids, central and portal veins (asterisks). (j, 10×) congestion of portal veins with fibroplasia (asterisk). (k, 10×; l, 20×) Liver sections of rats at 4^th^ week after albendazole with BMMSCs therapy showing: Slight congestion (asterisks) and portal area infiltrates with leukocytic without steatosis. (m, 20×) leukocytic infiltration (asterisk) in injured liver. (n, 10×) slight congestion of sinusoids and portal veins (asterisk) with leukocytic infiltrates (arrow). (o, 10×) nearly normal hepatic cells with leukocytic infiltrates of the portal vein. (p, 20×) dilatation and slight congestion of portal veins and leukocytic infiltration were observed in portal area. BMMSCs=Bone marrow mesenchymal stem cells.

Liver sections of healthy control rats provided nearly normal hepatocytes and central vein. On the other hand, liver sections of the untreated *T. vitulorum*-rat model showed congestion of sinusoids and portal veins with a wide area of hemorrhage with micro- and macro-vesicular steatosis. In addition, focal area of hepatic lytic necrosis with mononuclear infiltrates and biliary hyperplasia was also observed in the untreated *T. vitulorum*-rat model. Liver sections of rats in the 4^th^ week following albendazole therapy showed congestion of sinusoids, central and portal veins with hemorrhage, hepatic necrosis, and fibroplasia. BMMSCs therapy following albendazole administration (albendazole with BMMSCs) induced lymphocytic infiltration around the portal vein instead of monocyte infiltration in injured liver as a sign of immune response not a sign of inflammation. Slight congestion with nearly normal hepatic cells without steatosis or necrosis was observed in liver sections of rats in the 4^th^ week after albendazole with BMMSCs therapy (Figure-11).

### Biochemical studies

Measurement of total protein the sera of rats indicated that compared to the healthy control group there was a significant decrease in the untreated *T. vitulorum*-rat model. The level of total protein was significantly increased at 4^th^ weeks of albendazole with BMMSCs therapy comparing with the untreated *T. vitulorum*-rat model. In addition, the globulin concentration was significantly elevated (p<0.05) in the untreated *T. vitulorum*-rat model relative to the control. The activities of ALT, AST, and ALP were significantly elevated (p<0.01) in the untreated *T. vitulorum*-rat model relative to healthy control. However, the transplantation of BMMSCs with albendazole significantly declined the activities of these enzymes at the 2^nd^ and the 4^th^ weeks after albendazole with BMMSCs therapy (Table-4).

**Table-4:** Serum protein profile and liver enzymes activities in the serum of healthy control, treated and untreated *T. vitulorum*-rat models.

Parameters	Healthy control	*T. vitulorum-*rat models				

		Untreated	Albendazole		Albendazole with BMMSCs	
	
			2^nd^ week	4^th^ week	2^nd^ week	4^th^ week
Total protein (g/dL)	7.35±1.04	5.87^a^±0.45	6.15±0.91	6.37±1.04	6.89±1.07	7.23*±0.26
Albumin (g/dL)	3.87±0.27	3.84±0.07	3.82±0.28	3.53±0.47	3.72±0.86	3.85±0.63
Globulin (g/dL)	4.21±0.03	4.81^a^±0.03	4.85±0.73	4.29±1.05	4.25±0.33	4.18±0.35
A\G ratio	0.93±0.05	0.92±0.04	0.78±0.15	0.82±0.11	0.88±0.29	0.93±0.21
Cholesterol (mg/dL)	163.88±5.33	197.40^a^±4.86	191.76±7.23	187.12± 6.51	171.43±11.05	168.52*±7.81
ALT (U/L)	28.63±5.68	52.23^A^±4.72	49.32±8.59	46.58±6.18	33.74*±5.15	30.46*±6.01
AST (U/L)	130.83±11.03	241.77^A^±10.63	235.24±10.46	229.57±9.38	193.27*±9.14	156.18**±13.18
ALP (U/dL)	27.29±4.87	54.33^A^±4.83	51.11 ±6.17	48.29 ±4.36	48.41 ±7.50	32.11*±5.27

Mean±SE. Values marked with

a and

A are significantly different than their corresponding healthy control at p<0.05 and p<0.01, respectively. Values with

*, ** are significantly different at p<0.05 and p<0.01, respectively, relative to their corresponding untreated rats. BMMSCs=Bone marrow mesenchymal stem cells, *T. vitulorum=Toxocara vitulorum*, A\G=Albumin-Globulin, ALT=Alanine aminotransferase, AST=Aspartate aminotransferase, ALP=Alkaline phosphatase

### Leukogram, spleen lymphocytes viability, and phagocytic index

**Figure-12:**
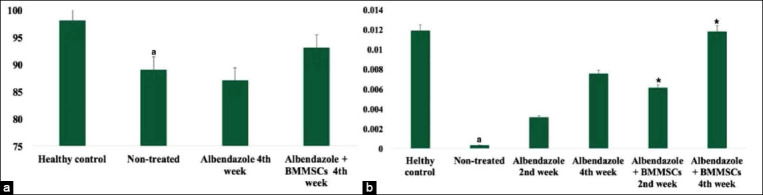
(a) Spleen lymphocytes viability % and (b) phagocytic index of different infected and treated groups. ^a^Significantly different relative to healthy control at p<0.01. *Significantly different relative to untreated at p<0.05. BMMSCs=Bone marrow mesenchymal stem cells.

Eosinophils and neutrophils count in the blood of the untreated *T. vitulorum*-rat model showed a significant elevation (p<0.05) relative to the healthy control. Groups treated with albendazole alone showed a significant decline in eosinophils % at 4^th^ week while groups treated with albendazole with BMMSCs therapy showed a significant decline in eosinophil and neutrophil levels at 2^nd^ and 4^th^ weeks (p<0.05). However, lymphocytes and N/L ratio significantly decreased (p<0.05) (Table-5). Spleen lymphocytes viability % of untreated *T. vitulorum*-rat model was 89%; however, it increased to 93% at the 4^th^ week after albendazole with BMMSCs transplantation (Figure-12a). The phagocytic index significantly decreased in the untreated *T. vitulorum*-rat model. However, it increased significantly (p<0.05) at the 4^th^ week in both albendazole treated group and albendazole with BMMSCs treated group compared to untreated *T. vitulorum*-rat model (Figure-12b).

**Table-5:** Eosinophils, neutrophils, lymphocytes, and N/L ratio of healthy control, treated and untreated *T. vitulorum-*rat models.

Parameters	Healthy control	*T. vitulorum-rat models*				

		Untreated	Albendazole		Albendazole with BMMSCs	
	
			2^nd^ week	4^th^ week	2^nd^ week	4^th^ week
Eosinophils %	2.86±0.11	7.62^A^±0.91	5.27±0.73	4.36*±0.58	3.74*±0.39	3.18*±0.85
Neutrophils %	71.23±3.21	84.47^a^±4.11	76.41±6.28	74.29±8.16	76.83±5.16	65.17*±4.21
Lymphocytes %	28.75±2.96	24.18±2.71	23.61±4.75	24.73±3.21	23.18±4.02	34.83*±3.29
N/L ratio	2.54± 0.37	3.63±0.51	3.31±0.06	3.08±0.12	3.35±0.12	1.87*±0.45

Mean±SE. Values having letter

a are significantly different than corresponding healthy control at p<0.05. Values having

* are significantly different than corresponding untreated at p<0.05. BMMSCs=Bone marrow mesenchymal stem cells, *T. vitulorum=Toxocara vitulorum*, N/L=Neutrophil/lymphocyte

In this study, serum cytokine levels (TNF-a, INF-γ, IL-4, and IL-10) were assessed in the healthy control, untreated and treated *T. vitulorum*-rat models. Serum concentrations of pro-inflammatory cytokines (TNF-a and INF-γ) were significantly increased (p<0.05) in the *T. vitulorum*-rat model than the healthy one. Besides, the anti-inflammatory cytokine (IL-4) was significantly decreased (p<0.05). After treatment with albendazole, no significant difference was observed in comparison to the untreated *T. vitulorum*-rat model. Transplantation of BMMSCs after albendazole treatment showed a significant decline in TNF-a and INF-γ associated with a significant elevation of IL-4 and IL-10 cytokines in the 2^nd^ and 4^th^ weeks of transplantation (Table-6).

**Table-6:** Serum cytokines profile of healthy control, treated and untreated *T. vitulorum*-rat models (ng/mL).

Parameters	Healthy control	*T. vitulorum-rat models*				

		Untreated	Albendazole		Albendazole with BMMSCs	
	
			2^nd^ week	4^th^ week	2^nd^ week	4^th^ week
TNF-a	9.270±1.38	15.382^a^±1.23	11.196±1.41	11.862±2.38	9.421*±1.83	9.220*±1.86
INF-g	8.481±1.11	14.663^a^±1.09	12.985±1.52	10.793±2.74	8.491*±1.33	9.045*±1.27
IL-4	12.363±1.51	6.735^a^±1.12	7.074±1.85	7.358±1.26	9.111±2.53	12.472*±2.01
IL-10	10.289±1.34	8.134±1.16	11.798 ±1.02	11.822 ±1.51	12.301*±1.04	12.613*±1.11

Mean±SE. Values marked with

a are significantly different than corresponding healthy control at p<0.05. Values having

* significantly different than corresponding untreated at p<0.05. BMMSCs=Bone marrow mesenchymal stem cells, *T. vitulorum*=*Toxocara vitulorum*, TNF=Tumor necrosis factor, INF=Interferon, IL=Interleukin

## Discussion

*T. vitulorum* is an intestinal nematode parasite of bovine species. The first aim of the present work was to study the manifestations of the *T. vitulorum*-rat model in the form of systemic immune responses and pathological aspects of the affected tissues with the kinetics of recovery after treatment. The therapeutic potential of combining BMMSCS transplantation after albendazole treatment was investigated as the second and the main objective of the present study.

Great interest has been focused on non-hematopoietic MSCs due to their potential for clinical cell therapy [39]. To correctly study this essential cell type, it is important to precisely define their viability, phenotype, and proliferation. In the present study, MSCs have been separated from rat bone marrow to adhere on the bottom of the culture dishes. BMMSCs continued to proliferate and propagate reaching confluency after 2 weeks from the initial seeding as reported in the study of Secunda *et al*. [40]. On initial seeding of cells, spindling began on the 2^nd^ day. The cell counting was 4.21×10^4^ cells/cm^2^ with 96.03±3.72% viability on reaching 80% confluence. This result was parallel to the finding of Abo-Aziza and Zaki [41]. Flow-cytometric analysis of the expression pattern of a variety of candidate markers has been used indicating positive markers, such as CD90 (82%), CD105 (79%), and negative markers CD34 (0.37%) and CD45 (0.42%). The same result was observed in the previous study [40]. The proliferation of BMMSCs was determined by means of BrdU, PD, and PDT assays. The percentage of BrdU positive cells was significantly increased on reaching 80% confluence. This result was reported previously [40]. The results of the PD revealed that BMMSCs showed maximal expansion potential in P3 and P4. This elevation continued until P9; however, it started to decline at P10 until the cell growth arrested in P16 and P17. Each PDT was analyzed in P1-P2, P5-P6, and P11-P12, respectively. In these passages, the PDT of P1-P2 and P5-P6 was shorter than P11-P12. Moreover, BMMSCs at 80% confluence showed 76.82±5.95 final PD score. These results collectively assure the candidacy of the isolated BMMSCs to be used for the therapeutic treatment of infected rats.

The selection of rats as a model for infection with *T. vitulorum*, as well as the planning day of sacrifice depended on the previous work of several investigators. Several experimental animal *T. vitulorum* models, including rabbits [42] and mice [12], were used in studying toxocariosis. However, the role of rats as an experimental model for the investigation of *T. vitulorum* infection was neglected in comparison to other laboratory animals [43]. However, in rodents, *Toxocara* larvae migrate throughout the organs for long periods. Therefore, experimental infection in rats might be a model for understanding animal toxocariosis. The selection of sacrifice was scheduled according to the previous studies on *Toxocara* tissue migration and the gradation of tissue damage [12].

In the present study, ultrasonography images showed multiple fibrotic foci in the liver of the untreated *T. vitulorum-*rat model. This area of fibrosis was also observed in the liver of rats at 2^nd^ and 4^th^ weeks following albendazole treatment. These results are following the gross changes in the study of Atallah *et al*. [12]. However, BMMSCs transplantation after albendazole treatment regenerated the healthy liver appearance at 2^nd^ and 4^th^ weeks. These findings indicated that alterations in liver features due to *T. vitulorum* could persist after treatment by albendazole. However, the liver of *T. vitulorum* rat model could be regenerated by BMMSCs transplantation. Several studies have demonstrated the beneficial effects of MSCs on the regeneration of injured tissues [19,20].

The macroscopic examination in this study after sacrifice showed hepatomegaly with severe congested dark borders of the liver of the untreated *T. vitulorum*-rat model. Similarly, hepatomegaly congested liver following albendazole administration was observed. On the other hand, the normal appearance of the livers with approximately normal size was observed in rats at the 4^th^ week after albendazole with BMMSCs therapy. Microscopically, *T. vitulorum* infection resulted in congestion of sinusoids and portal veins with a wide area of hemorrhage and micro and macrovesicular steatosis. These data were in agreement with the findings of Atallah *et al*. [12] in mice. Focal area of hepatic lytic necrosis with mononuclear infiltrates and biliary hyperplasia was also observed in the untreated *T. vitulorum-*rat model that was parallel to the findings of Kumar *et al*. [44] who attributed the changes to the immune response against *T. vitulorum* infection. Several investigators studied the migration of *Toxocara* larvae in mice [12,13]. The recorded lesions were seen in different organs and represented by coagulative necrosis induced by larval migration, besides aggregation of inflammatory cells, mainly eosinophils that were replaced by macrophages and finally fibroblasts. In the present study, central vein dilatation and hemorrhage in the liver at the 4^th^ week following albendazole treatment were noted that may be effect of the longtime of infection or the production of metabolic byproducts of the parasite. Another explanation could be the effect of the immune response in the form of acidophil, monocyte, and factor - a and b production was the reason for hepatic central vein expansion by parasitic infection [45]. However, BMMSCs therapy following albendazole administration induced lymphocytic infiltration instead of monocyte in the injured liver as a sign of immune response, not a sign of inflammation. Besides, this therapy was resulted in nearly healthy hepatic cells with lymphocytic infiltrates of the portal vein.

Serum protein profile and liver enzyme activities were assessed in the present study. Total protein was significantly decreased in the untreated *T. vitulorum-*rat model relative to the healthy control group. The level of total protein was significantly increased at 4^th^ week of albendazole with BMMSCs therapy comparing with the untreated *T. vitulorum-*rat model. Furthermore, a significant elevation in the globulin concentration was noticed in the untreated *T. vitulorum-*rat model relative to the healthy control. The levels of liver enzymes were significantly elevated in the untreated *T. vitulorum-*rat model relative to healthy control. However, transplantation of BMMSCs with albendazole significantly declined the release of liver enzymes at the 2^nd^ and the 4^th^ weeks after albendazole with BMMSCs therapy that indicating improved liver cellularity. These biochemical, macroscopical, and histopathological findings confirmed the ultrasonographic results.

Data obtained on the status of immunity following infection or treatment with anthelmintic drugs is conflicting. However, the available data on cell-mediated and humoral immune status are rare. Thomas *et al*. [46] stated two-contradictory hypothesis that antibodies against *T. vitulorum* antigens lack sensitivity and specificity to detect infection due to high cross-reaction with other nematode antigens, or highly specific antibodies to *T. vitulorum* antigens. The present work primarily monitored the humoral immunomodulation by measurement of the levels of *T. vitulorum* anti-Tv-ES, Tv-CS, Tv-E, Tv-DTC, and Tv-C antibodies in the serum of *T. vitulorum* rat model after infection and treatment. In this work, *T. vitulorum* infection resulted in an elevation in immunoglobulins titers against all *T. vitulorum* antigens used relative to the control. This result agreed with the finding of Souza *et al*. [47] in bovine sera against *T. vitulorum* infection during lactation and pregnancy. However, treatment with albendazole alone resulted in a significant drop in the antibody level against Tv-CS, Tv-C, and Tv-DTC parasite antigens in the 2^nd^ week post-treatment. These results were parallel to the same previous study [47] in naturally infected buffalo that developed immunity and keep high levels of antibodies against *T. vitulorum* antigens and these antibodies are transferred to their calves through the colostrum. However, transplantation of BMMSCs after treatment with albendazole resulted in a significant drop in antibody level against two parasite antigens (Tv-C and Tv-DTC) in the 2^nd^ week post-treatment. This drop became more pronounced and significant at the 4^th^ week post-treatment against all prepared *Toxocara* antigens in rats transplanted with BMMSCs. Immunization trials with different *T. vitulorum* antigens using different experimental animals were investigated by some authors. Amerasinghe *et al*. [48] reported that the perienteric fluid antigen (PeAg) induced 100% protection in mice when compared with ESAg, which produced slightly lower protection (82.6%). Furthermore, vaccination of rabbits by execratory/secretory antigen (EEAg) decreased tissue invasion ability of larvae [49]. The study of Mahdy *et al*. [50] demonstrated the value of rats as an experimental model for investigating *T. vitulorum* infection. It characterized PeAg as a valuable immunogenic and protective antigen to minimize the infection by *T. vitulorum* between mother and calves in infected farms. In addition, infected mice showed elevated levels of *Toxocara* specific immunoglobulin G that persisted up to 2 months post-infection [51]. In the present study, the cellular immune picture was monitored by measurement of leukocytes count, spleen lymphocytes viability, phagocytic index, and Th1/Th2 cytokines. Helminthic infections of human and laboratory animals including *T. vitulorum* induced a variety of cell-mediated immune responses such as tissue eosinophilia and mastocytosis [52].

The percentage of eosinophils and neutrophils in the blood of the untreated *T. vitulorum-*rat model showed a significant elevation relative to the healthy control. The model treated with albendazole alone showed a subsequent decline in eosinophil levels at 4^th^ week while model treated with albendazole with BMMSCs therapy showed a significant decline in eosinophils and neutrophil levels at 2^nd^ and 4^th^ weeks. As such, these cells seemed to play an essential role during innate anti-parasite responses. In addition to the decrease in neutrophils, similar responses in the hematological profile were reported previously [51]. However, lymphocytes % and N/L ratio was found to be significantly decreased. Viability of spleen lymphocytes from untreated *T. vitulorum-*rat model was 89%. However, it increased to 93% at the 4^th^ week after albendazole with BMMSCs transplantation. The phagocytic index significantly decreased in the untreated *T. vitulorum-*rat model. However, it increased significantly at 4^th^ week in both albendazole treated group and albendazole with BMMSCs treated group comparing to the untreated *T. vitulorum-*rat model. The obtained data were indicated an improved cell-mediated immune response after albendazole with BMMSCs transplantation.

During parasitic disease, cytokines produced from different immune cells and play a central role in disease outcome [53]. However, the cytokines function in both local and even remote distant ways was to regulate the immune response [54]. Cytokines balance can be considered as a measure of disease progression and/or host protection. In chronic toxocariosis, the superiority of Th2 was reported [52]. However, the reason for switching of dominance from Th1 to Th2 is unknown. Therefore, one of the goals of this work was to discover the correlation between alteration in cytokines caused by toxocariosis and beneficial effects of BMMSCs transplantation on the direction of cytokine balance to be useful for recovery. Parasitic infections are controlled by cells producing Th1/Th2 cytokines [55]. Cytokines such as TNF-α, IL-6, and IFN-γ have protective inflammatory responses for the development of tissues fibrosis and granulomatous changes [56]. The previous studies have recorded that different mechanisms control the cytokines secretion in *Toxocara* infected animals. Another study showed that cytokines produced from macrophages might be due to the result of stimulation of *Toxocara*-derived antigens to enhance the differentiation of Th2 cells [57]. A directly proportional relationship was observed between Th1 cytokines and *Toxocara* larvae migration [51]. Various safety mechanisms coordinate the following steps of the inflammation. The metabolites of *Toxocara* can activate the release of Th2 cytokines [51]. Tv-ES of *toxocara* can activate numerous cells for cytokines production and immune homeostasis contribution [58]. This shifting was accompanied by the production of IL-10 [59]. A close relationship has been reported between eosinophil levels and the production of Th2 lymphocytes [59]. The serum concentrations of pro-inflammatory cytokines (TNF-α and INF-γ) were significantly increased (p<0.05) in the untreated *T. vitulorum*-rat model than the healthy one. Besides, the anti-inflammatory cytokine (IL-4) was significantly decreased (p<0.05). After treatment with albendazole, no significant difference was observed in comparison to the untreated *T. vitulorum-*rat model. However, transplantation of BMMSCs after albendazole treatment showed a significant decline in TNF-a and INF-γ associated with a significant elevation of IL-4 and IL-10 cytokines in the 2^nd^ and 4^th^ weeks of transplantation. The higher levels of TNF-α and INF-γ observed might be a result of *T. vitulorum* infection. Parallel with the pro-inflammatory processes, a suppressive, IL-10 was also elevated after BMMSCs transplantation and it might be due to the secretion of regulatory T cells (Treg) that suppress fibrosis and chronic inflammatory response to the parasite [59]. The transplantation of BMMSCs might trigger higher eosinophil levels that could activate Treg cells, which additionally produce IL-10 release. The previous data were parallel in the observations of the experimental model of murine toxocariosis infection [60]. BMMSCs enhance the release of IL-10 cytokine by Treg cells in *Toxocara* infection [54]. BMMSCs transplantation induced IL-4 cytokine. Shooting of the Th2 (IL-4) response normally associated with the down-regulation of Th1 (TNF-α) response. These results confirmed that BMMSCs could modulate the immune system that orchestrated a careful balance between cytokines and immune regulatory responses to function effectively against *T. vitulorum* infection. Following a significant elevation in eosinophils% and neutrophils% in untreated *T. vitulorum-*rat model and decreased in albendazole with BMMSCs treated model, we discovered dominance of Th2 cytokines. However, this dominance and the elevation leukocytes seemed to occur after the larval migration through the hepatic tissues. This evidenced by the absence of granulomatous formation or presence of larvae in the tissues [61]. Another evidence is that the production of antibody with the shifting to Th2 response that occurred during the late stage of infection has been reported [62]. These authors also found elevated levels of IL-10 in the lungs at 1-2 weeks’ post-infection, we did not find elevated IL-10 concentrations in serum of untreated *T. vitulorum-*rat model throughout this experiment, indicating that the production of this cytokine occurred only after stem cell therapy and this elevation was attributed to the immunomodulatory effect of the transplanted BMMSCs.

## Conclusion

The final results indicated that the liver functions, histological architecture, and immune parameters were aggravated following the experimental infection of rats by *T. vitulorum*. Co-Adjuvant therapy of BMMSCs to albendazole treatment can regenerate injured hepatic tissue. Besides, it can orchestrate host defensive immune responses against *T. vitulorum* antigens. We concluded that this work could define more clearly the events that manipulate the host immune, histopathological, and biochemical responses to minimize obstacles in using stem cell therapy in human and animal toxocariasis.

## Authors’ Contributions

FAMA conceived and designed the study. FAMA performed the isolation and characterization of BMMSCs including cell counting, viability, CFUs and PD assays, AAZ contributed to phenotyping and BrdU assay. AIA and SMA performed the experiments contributed to preparation of *T. vitulorum* antigens, preparation, and potency assay of immunoglobulins and *T. vitulorum-*rat model. FAMA interpreted ultrasonographic results and perform the biochemical analysis. AIA and SMA performed histopathological examination. AIA and SMA assisted the humoral and cell-mediated immune response and leukogram. FAMA performed spleen lymphocytes viability, phagocytic index and Th1/Th2 cytokines. AAZ, AIA, and SMA performed the statistical analysis. FAMA and AAZ wrote the paper. AIA and SMA reviewed the paper. All authors have read and approved the final manuscript.
